# An Approach for Differential Diagnosis of Breast Tumors by ctDNA Methylation Sequencing

**DOI:** 10.1002/cam4.71004

**Published:** 2025-06-20

**Authors:** Xianyu Zhang, Yanling Yin, Zhujia Ye, Xingda Zhang, Wei Wei, Yi Hao, Liuhong Zeng, Ting Yang, Dalin Li, Jun Wang, Dezhi Zhao, Yanbo Chen, Shan Lei, Yongdong Jiang, Youxue Zhang, Shouping Xu, Abiyasi Nanding, Yajie Gong, Siwei Li, Yuanyuan Yu, Shilu Zhao, Siyu Liu, Yashuang Zhao, Zhiwei Chen, Shihui Yu, Jian‐Bing Fan, Da Pang

**Affiliations:** ^1^ Department of Breast Surgery Harbin Medical University Cancer Hospital Harbin China; ^2^ AnchorDx Medical Co., Ltd. Guangzhou China; ^3^ Department of Breast Surgery Zhujiang Hospital, Southern Medical University Guangzhou China; ^4^ Department of Pathology Harbin Medical University Cancer Hospital Harbin China; ^5^ Department of Epidemiology Harbin Medical University Harbin China; ^6^ AnchorDx, Inc. California California USA; ^7^ Guangzhou Kingmed Center for Clinical Laboratory Co., Ltd. Guangzhou China; ^8^ Guangdong‐Hong Kong‐Macao Joint Laboratory of Respiratory Infectious Disease Guangzhou Guangdong China; ^9^ Guangdong Provincial Key Laboratory of Genetic Disease Diagnostic Guangzhou Guangdong China; ^10^ Department of Pathology, School of Basic Medical Science Southern Medical University Guangzhou China; ^11^ Department of Biochemistry and Molecular Biology, School of Basic Medical Sciences Southern Medical University Guangzhou China; ^12^ Department of General Surgery Zhujiang Hospital, Southern Medical University Guangzhou China

**Keywords:** breast cancer, breast ultrasonography, core needle biopsy, ctDNA, mammography, methylation

## Abstract

**Background:**

Breast ultrasonography and mammography remain predominant in breast tumor evaluations, yet they often result in false positives, particularly for tumors classified as BI‐RADS 4a or those no more than 10 mm, which are not ideal for core needle biopsy (CNB). Early‐stage breast cancer detection via circulating tumor DNA (ctDNA) methylation holds potential to bridge these diagnostic gaps.

**Methods:**

We curated a breast cancer‐specific panel by harnessing methylation profiles from in‐house and public databases. Leveraging breast tissue‐plasma‐leukocyte samples, we identified breast cancer‐specific markers, culminating in a 103‐marker methylation model which underwent rigorous validation in two independent cohorts. To assess its performance, we compared it against the accuracy of ultrasonography, mammography, and CNB.

**Results:**

The 103‐marker model exhibited remarkable proficiency in discerning benign from malignant breast tumors in plasma, with AUCs of 0.838, 0.838 and 0.823 in the validation set and two independent test sets, respectively. In BI‐RADS 4a breast cancer, when compared to ultrasonography or mammography, the model augmented breast cancer diagnostic accuracy by 40.58% and 25.49%, separately. Retrospective analyses suggested that our model achieved a sensitivity of 66.67% (4/6) and a specificity of 80.36% (45/56) for surgical patients in the BI‐RADS 4a category with tumors ≤ 10 mm, who did not undergo CNB, potentially sparing 45 benign patients from overtreatment. Notably, significant differences emerged in cancer scores between DCIS and invasive ductal carcinoma (*p* < 0.05). Higher cancer scores correlated with a more unfavorable prognosis (*p* < 0.05).

**Conclusions:**

The 103‐marker methylation model demonstrates impressive performance in distinguishing between malignant and benign tumors, facilitating precise early diagnosis of BC, and holds promise as a prognostic tool.

## 1 Introduction

Breast cancer (BC) is the most common malignant tumor in women worldwide [1]. There is an unlimited demand for its accurate differentiation from benign breast tumors given that ultrasonography Breast Imaging‐Reporting and Data System (BI‐RADS) subcategories 4a and 4b have positive predictive values (PPVs) of only 6% and 25%, respectively [2]. First, the differentiation between malignant and benign tumors can reduce overtreatment (such as for benign tumors classified with BI‐RADS 4a or higher findings [3]), further reducing the burden on the health care system. Second, studies to distinguish benign from malignant lesions may lead to the emergence of more sensitive tests for early breast cancer (stage I or even stage 0). For example, the current detection of ductal carcinoma in situ (DCIS), which has a relatively indolent behavior, leads to a particular dilemma in balancing the risk of causing unnecessary overtreatment or leaving an increased risk of progression [4]. In view of the higher false‐negative rate of core needle biopsy (CNB) for smaller BC tumor size, most patients of this type choose incisional biopsy under general anesthesia, which costs at least ten thousand yuan in China. As a result, it is particularly important to develop a noninvasive detection method with high accuracy (ACC) among patients with breast diseases that helping to reduce the burden of BC and to relieve the anxiety of patients.

Liquid biopsies possess the capability to identify, profile, and track cancers more swiftly and with minimal invasiveness compared to conventional methods [5].

They are utilized as biomarkers for cancer screening, diagnosing, monitoring therapeutic responses, and predicting prognosis [6, 7]. The integration of circulating tumor DNA (ctDNA) with Breast Imaging‐Reporting and Data System (BI –RADS) scores enhances the accuracy of early‐stage breast cancer diagnostics. For patients with a BI‐RADS classification of ≥ 4a, this combined approach yields a positive predictive value of 92.45%, a sensitivity of 74.24%, and a specificity of 92.00%, demonstrating a significant improvement over the BI‐RADS system alone [8]. However, ctDNA mutation detection faces limitations due to the finite number of detectable somatic mutations and the associated detection threshold (LoD). Additionally, it is susceptible to interference from copy number variations and non‐tumor genetic changes, such as clonal hematopoiesis (CH). Furthermore, prior research indicates that 30%–50% of BC patients with stage I‐III cancers, and 15%–20% of those with stage IV cancers, do not present detectable somatic mutations [9]. DNA methylation changes are present in almost all cancers and occur at precancerous or early cancer stages, making them considered ideal markers for the early diagnosis of cancer [10]. In our earlier studies, we employed the MethyLight method [11], and a more recent study used whole genome bisulfite sequencing (WGBS) [12] for diagnosing early‐stage breast cancer. However, neither method could simultaneously meet the requirements for genomic coverage and sequencing depth. Recent study indicates that DNA methylation alterations in peripheral blood mononuclear cells (PBMCs) could be used as markers for early detection of bc [ 13]. These investigations have demonstrated that ctDNA methylation has the potential to serve as a complementary cancer biomarker.

Therefore, a new technique (such as targeted methylation sequencing) is urgently needed to compensate for the above deficiencies and further improve the differentiation of benign and malignant breast tumors.

The Circulating Cell‐free Genome Atlas study (CCGA) is the most successful early‐stage diagnosis study to date based on cell‐free DNA (cfDNA) sequencing. However, its sensitivity is low for early‐stage cancer (stage I, 16.8%) [14]. Optimizing sequence methods and improving research design are important ways to overcome this drawback. In this study, we adopted the targeted methylation sequencing method, which has great advantages in the clinical limit of detection (LOD) [15], developed a new BC‐specific panel, and utilized plasma samples from patients with both benign and malignant breast tumors on the basis of a previous study of breast cancer screening [16] (patient and healthy control design, sensitivity 75% for stage I breast cancer) to establish a BC‐specific 103‐marker methylation model for differentiating benign and malignant breast tumors. Additionally, we validated these 103 plasma‐specific markers using 265 tissue samples, confirming that these markers also perform well in tissue samples. The final performance of our model was evaluated in two independent cohorts: 88 plasma samples (with a benign to malignant ratio of 46:42) and 106 plasma samples (with a benign to malignant ratio of 46:60). This model not only improves the early detection rate of breast cancer but also represents the first successful differentiation of benign and malignant breast tumors using targeted methylation sequencing.

## 2 Results

### 2.1 Development and Verification of a Diagnostic Model for Malignant and Benign Breast Tumors

The breast cancer‐specific methylation panel consists of markers from the AnchorDx database (81%), The Cancer Genome Atlas (TCGA) (10%) and other sources (9%), including 13,676 differentially methylated regions (DMRs) consisting of 129,794 3‐CpG markers.

**FIGURE 1:**
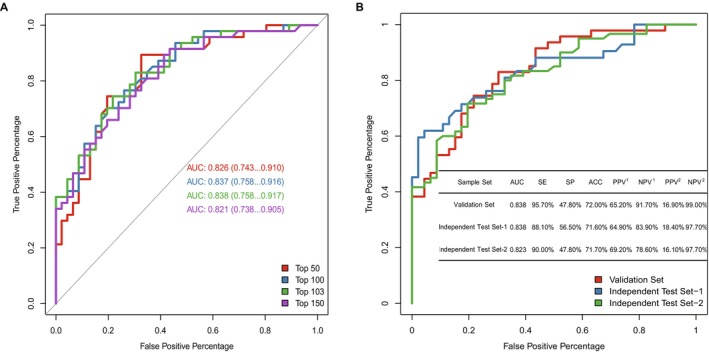
Development of the diagnostic model for malignant and benign breast tumors and verification of its diagnostic performance in two independent test sets. (A) ROC curves of different top marker combinations in the validation set (*n* = 93). (B) ROC curves, SE, SP, ACC, PPV, and NPV of the 103‐marker model in the validation set and two independent test sets (*n* = 194). SE, sensitivity; SP, specificity; ACC, accuracy; NPV, negative predictive value; PPV, positive predictive value; ROC curve, receiver operating characteristic curve [1], these values were calculated by the clinical prevalence of enrolled patients (50%) [2], these values were calculated by the clinical prevalence in the real world (10%).

The methylation profiles of 307 enrolled plasma samples (training set: validation set = 214:93) and markers selected from 2272 bc‐specific markers were applied for model development (Figure S1). Performing a comparison of the models consisting of the top 50, 100, 103, or 150 markers, we selected a model including the top 103 markers since it showed the strongest power in differentiating malignant and benign breast tumors, with an area under the curve (AUC) of 0.838 (95% CI: 0.758–0.917) (Figure 1A). In addition, the 103‐marker model presented great diagnostic power for breast cancer in tissue samples, with an AUC of 0.997 (95% CI: 0.992–1) (Figure S4). Moreover, the cancer scores of malignant tumors were significantly higher than those of benign tumors in both breast tumor tissues and plasma (Figure S5). This finding indicates that the methylated signals of these 103 markers are consistent between breast tumor tissues and plasma.

Upon conducting Gene Ontology (GO) and Kyoto Encyclopedia of Genes and Genomes (KEGG) pathway analyses on the 103 methylation markers, corresponding to 76 genes, we discovered that these genes are crucially involved in key biological processes. These include G protein‐coupled receptor signaling (e.g., CRHR2, GHSR, GNG7, HTR1B, NPY, RGS22) [17, 18, 19, 20, 21, 22, 23], transcription regulation via DNA‐binding (e.g., TLX2, IRF4, NEUROD2, PAX9, IKZF3, RAX, PTF1A, LHX1) [24, 25, 26, 27, 28, 29, 30], responses to growth factors (e.g., RUNX3, GCNT2, LHX1, PAX9, CHRD, SHISA2) [31, 32, 33], cell proliferation (e.g., CDK2AP1) [34, 35], and hormone signaling (e.g., RUNX3) [36, 37]. The aberrant methylation patterns of these markers suggest their significant roles in the initiation and progression of breast cancer (Tables S1 and S2).

With the expectation of clinically applying the methylation model, to detect as many positive patients as possible, a cutoff for the 103‐marker model with a sensitivity (SE) above 95% and negative predictive value (NPV) above 90% was chosen. The 103‐marker model achieved an overall SE of 95.7% at a specificity (SP) of 47.8% in the validation set under this cutoff, and its NPVs were 91.7% and 99% under the current prevalence (50%) and the real clinical prevalence (10%), respectively (Figure 1B). For different pathological stages, the sensitivities were 100%, 86.67%, 100% and 100% for stages 0, I, II, and III, respectively. In addition, this model also had a higher SE for different molecular subtypes. The sensitivities of diagnosing human epidermal growth factor receptor 2 ‐positive (HER2+) breast cancer and triple‐negative breast cancer (TNBC) were 100%, and for the hormone receptor‐positive (HR+)/HER2‐negative (HER2−) subtype, they were 93.1% (Table 1).

**TABLE 1:** Performance of the methylation model in different cancer stages and different molecular subtypes in the validation set and independent test sets.

	Validation set (*n* = 93)					Combined independent test sets (*n* = 194)				
	Negative	Positive	Total	Sensitivity	Specificity	Negative	Positive	Total	Sensitivity	Specificity
Stage										
0	0	3	3	100.00%	—	1	6	7	85.71%	—
I	2	13	15	86.67%	—	6	22	28	78.57%	—
II	0	22	22	100.00%	—	4	54	58	93.10%	—
III	0	7	7	100.00%	—	0	9	9	100.00%	—
Molecular subtypes										
HER2+	0	8	8	100.00%	—	1	25	26	96.15%	—
HR+/HER2 —	2	27	29	93.10%	—	8	49	53	92.45%	—
TNBC	0	5	5	100.00%	—	1	13	14	92.86%	—
Unknown	0	5	5	100.00%	—	1	4	5	80.00%	—
Total	2	45	47	95.74%	—	11	91	102	89.22%	—
Benign	22	24	46	—	47.83%	48	44	92	—	52.17%

To verify the performance of the 103‐marker model, 194 plasma samples were used and assigned to two independent test sets, Test‐1 (malignant: benign = 42: 46) and Test‐2 (malignant: benign = 60: 46). This model had stable predictive power with AUCs of 0.838 (95% CI: 0.752–0.924) and 0.823 (95% CI: 0.746–0.900) in the two independent test sets (Figure 1B) as well as high detection rates for different BC subtypes (HER2+, 96.15%; HR+/HER2−, 92.45%; TNBC, 92.86%) (Table 1). Moreover, the SE of the model increased with the pathologic stage (stage 0: 85.71%; stage I: 78.57%; stage II: 93.1%; stage III: 100%). The cancer scores [38] of different subtypes of malignant tumors in stages 0—I and II—III were significantly higher than those of benign tumors (Figure S6).

### 2.2 Comparison of Breast Cancer Diagnostic Performance Among Mammography, Breast Ultrasonography, and the Methylation Model

Compared to breast ultrasonography and mammography, the accuracy (ACC) of the methylation model in breast cancer diagnosis was obviously higher (71.65%, 139/194) than breast ultrasonography (64.43%, 125/194) and was slightly lower (72.19%, 135/187) than mammography (75.94%, 142/187) (Table S3). For malignant tumors in stages 0–I, although breast ultrasonography (98.11%, 52/53) was more sensitive than mammography (80.77%, 42/52) and the methylation model (83.02%, 44/53) (Table 2), its specificity was relatively low (26.09%, 24/92) (Table S3).

**TABLE 2:** Comparison of the performance in the diagnosis of breast cancer stages among the methylation model, breast ultrasonography, mammography, and combined model in validation and two independent test cohorts.

Cohort	Detection	Breast ultrasonography	Methylation model	Mammography	Methylation model
0, I	SE	98.11% (52/53)	83.02% (44/53)	80.77% (42/52)	84.62% (44/52)
II	SE	98.75% (79/80)	95.00% (76/80)	92.31% (72/78)	94.87% (74/78)
III	SE	93.75% (15/16)	100.00% (16/16)	86.67% (13/15)	100.00% (15/15)

In comparison of performance on BI‐RADS 4 category (Test‐1 and Test‐2, *n* = 144), the methylation model presented obvious advantages in that it increased the ACC by 18.05% (70.83%, 102/144 vs. 52.78% 76/144) on the basis of breast ultrasonography; it also improved the ACC by 8.85% (78.76%, 89/113 vs. 69.91%, 79/113) on the basis of mammography (Table 3). The performance of the methylation model was better than that of both breast ultrasonography (ACC: 57.97%, 40/69 vs. 17.39%, 12/69) (Test‐1 and Test‐2, *n* = 69) and mammography (ACC: 68.63%, 35/51 vs. 43.14%, 22/51) (Test‐1 and Test‐2, *n* = 51), especially for the BI‐RADS 4a subcategory (Table S4). Patients with this subcategory have a low proportion of breast cancer and tend to be in early stages [39]. These results indicated that the methylation model was superior to ultrasonography and mammography in the diagnosis of early breast cancer.

**TABLE 3:** Comparison of the performance in the diagnosis of BI‐RADS 4 breast cancer among breast ultrasonography, mammography and the combined models.

Cohort	Detection	Ultrasonography	Methylation	Combined (*U* + *M*)	Mammography	Methylation	Combined (*M* + *M*)
Validation set	ACC	49.18% (30/61)	70.49% (43/61)	70.49% (43/61)	66.67% (28/42)	71.43% (30/42)	64.29% (27/42)
Independent test set‐1 and 2	ACC	52.78% (76/144)	70.83% (102/144)	71.53% (103/144)	69.91% (79/113)	78.76% (89/113)	69.91% (79/113)

### 2.3 Performance of the Combination of Breast Cancer‐Specific Methylated Markers and Breast Ultrasonography or Mammography

Using the combination of the 103‐marker model and breast ultrasonography, the overall accuracy (78.35%, 152/194) of the combined model was much greater than applying either breast ultrasonography (64.43%, 125/194) or the methylation model (71.65%, 139/194) alone (Table S3). When the SE was above 90%, the overall SP of the combined model (58.70%, 54/92) was 32.61% higher than that of breast ultrasonography (26.09%, 24/92) (Table S3). In particular, among the patients diagnosed with BI‐RADS category 4, more negative patients were found using the combined model (90.91%, 30/33) than using either of them alone (79.55%, 35/44) (Table 3). The combined model (56.52%, 39/69) and the methylation model (57.97%, 40/69) had similar accuracy in the diagnosis of breast cancer in the BI‐RADS 4a subcategory (Table S4). Moreover, there was no significant improvement from the combination of the 103‐marker model and mammography compared to using mammography or the methylation model alone. These findings demonstrated that the use of the 103‐marker model in combination with breast ultrasonography could better promote the accuracy in breast cancer diagnosis than mammography, especially for women at higher risk ages (≥ 40 years old) (Figure S7); meanwhile, the breast cancer‐specific methylated markers contributed the most to the combined model.

### 2.4 Comparison of Breast Cancer Diagnostic Performance Between the Methylation Model and Ultrasound‐Guided Core Needle Biopsy

**FIGURE 2:**
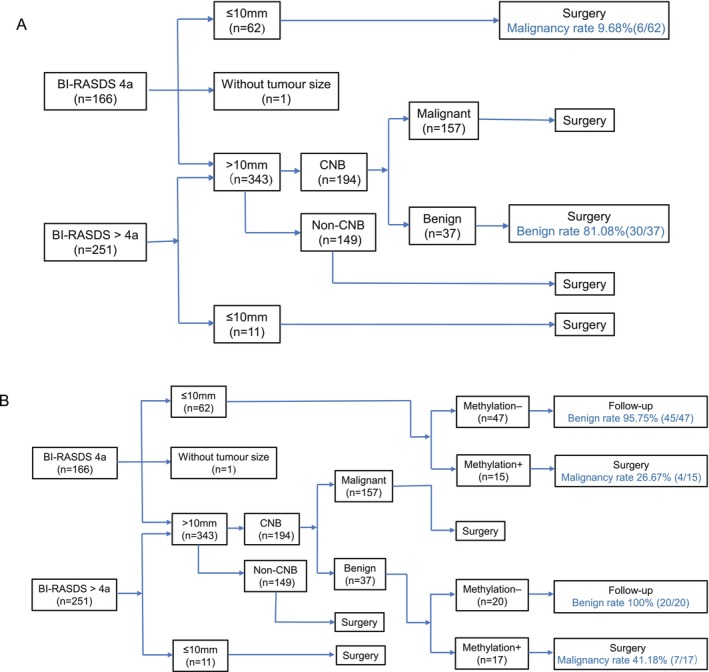
Workflow of the clinical diagnosis and retrospective analysis by the methylation model. (A) Process of clinical diagnosis and treatment. (B) Retrospective analysis by the methylation model. BI‐RADS, Breast Imaging‐Reporting and Data System; CNB, core needle biopsy. Methylation +, positive results detected by the methylation model; Methylation −, negative results detected by the methylation model. Benign rate = actual number of benign patients/total number of patients; malignancy rate = actual number of malignant patients/total number of patients.

Core needle biopsy (CNB) is one of the main diagnostic methods for suspicious lesions and is usually conducted under the guidance of breast ultrasonography. We compared the methylation model and CNB (Figure 2). Tumor size is an important factor to consider when we choose the means of diagnosis and therapy for patients with breast ultrasonography BI‐RADS category 4a or above.

If the tumor diameter was no more than 10 mm, the patients were more likely to undergo surgery directly since the tumors were too small for CNB. In this study, the malignancy rate was only 9.68% (6/62) in BI‐RADS 4a tumors no more than 10 mm. In a retrospective analysis of these patients, the methylation model increased the malignancy rate to 26.67% (4/15), with the true benign rate reaching 95.75% (45/47) in follow‐up patients, excluding unnecessary surgery.

If tumors were greater than 10 mm in BI‐RADS 4a or above categories (*n* = 343), CNB was a necessary diagnostic method (*n* = 194). In this study, 37 patients with negative CNB results underwent surgeries due to inconsistent results with image findings, and 18.9% (7/37) of them were detected as having malignancies postoperatively. While evaluating by a retrospective analysis, we detected 17 of the CNB‐negative patients as positive by the methylation model, including all 7 false‐negative patients; 20 of them were detected as negative, and they were all true benign patients. Thus, the methylation model had the potential to increase the negative prediction rate and decrease unnecessary surgeries for indeterminate breast tumors.

### 2.5 Methylation Signal Changes in Patients With Different Tumor Lesions

**FIGURE 3:**
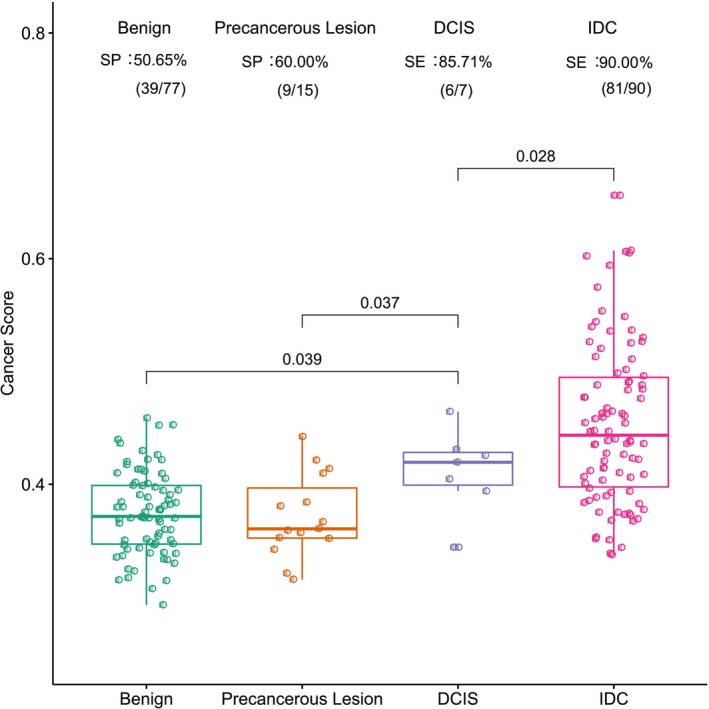
Distribution of the methylation signals in different pathological types of breast lesions. This figure illustrates the distribution of cancer scores predicted by the 103‐marker methylation model across various pathological types of breast lesions, including Benign, Precancerous Lesion, Ductal Carcinoma In Situ (DCIS), Invasive Ductal Carcinoma (IDC), and Other Malignant. The *x*‐axis categorizes the types of breast lesions, while the *y*‐axis quantifies the cancer scores. Statistical analysis was conducted using a *t*‐test, with each group compared to the DCIS group. The detection rate—the ratio of detected patients to the total number of patients within each pathological type—is displayed above each boxplot. Statistical significance is indicated by a *p*‐value less than 0.05.

To investigate the differences in methylation signals among different breast diseases, we evaluated the performance of the 103‐marker model on breast lesions, such as benign, precancerous, ductal carcinoma in situ (DCIS), and invasive ductal carcinoma (IDC) lesions, in the combined independent test sets. The results showed that the cancer scores of DCIS lesions were significantly higher than those of benign and precancerous lesions, while they were obviously lower than those of IDC (*t* test, *p* < 0.05) (Figure 3).

### 2.6 Correlation Between Methylation Signals and Postoperative Recurrence or Metastasis

**FIGURE 4:**
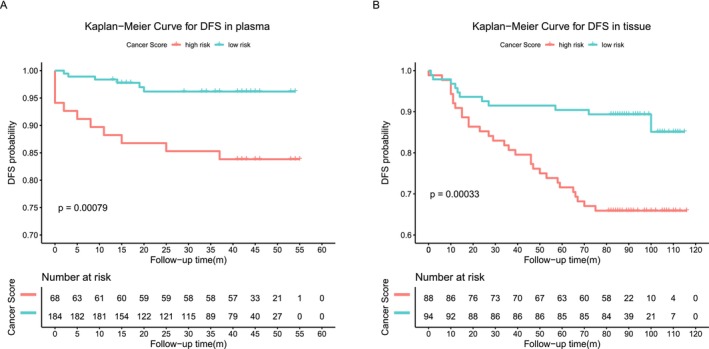
Cancer score of the methylation model predicts the prognosis of patients with breast cancer. (A) Kaplan–Meier curves show the disease‐free survival (DFS) of 252 plasma samples. (B) Kaplan–Meier curves show the disease‐free survival (DFS) related to 182 tissue samples stratified by the cancer score. The cutoff of the plasma was 0.83 (95% CI: 0.08–0.57), and the cutoff of the tissue was 0.9905 (95% CI: 0.15–0.6).

To evaluate the correlation between the methylation signals of the 103‐marker model and postoperative recurrence or metastasis, we analyzed 182 tissue samples from the independent tissue set and 252 plasma samples that had complete follow‐up information (Table S5). We used univariate and multivariate regression analyses to assess the impact of cancer scores and clinicopathological features on the prognosis of these patients. Both univariate and multivariate regression analyses showed that the cancer score of the methylation model was an independent adverse prognostic factor in plasma. In tissue, the cancer score was identified as a significant prognostic predictor by univariate analysis (Tables S6 and S7). Breast cancer recurrence or metastasis occurred in patients with higher cancer scores and shorter disease‐free survival (DFS) (*p* = 0.00079, *p* = 0.00033) regardless of whether it was detected using plasma or tissues. This result indicated that the methylation model had the potential to distinguish between malignant breast tumors with a low risk of recurrence or distant metastases and those with a high risk (Figure 4A,B and Figure S8).

## 3 Discussion

Early detection of breast cancer can greatly improve the patient survival rate, and the rate maintains an increasing trend year by year due to imaging examination, which contributed to an improvement in the 5‐year survival rate of BC from 73.1% to 82% during 2003–2015 [40]. When the diameter of solid tumors reaches 1–2 mm, neovascularization can be observed in imaging examination [41], but it is still difficult to differentiate benign and malignant tumors with diameters ≤ 10 mm via ultrasonography [42]. Even CNB exhibited a high false‐negative rate for that kind of tumor in previous studies (7.8% ~ 16.2%) [43, 44]. CNB involves many steps and may cause loss of tumor tissues as well as destruction of tumor integrity, all likely resulting in an underestimated diagnosis and difficulties for postoperative pathology [45]. In addition, the accuracy of core needle biopsy can be reduced by the pathological type of tumors, such as fibrocystic condition, atypical cell cluster, necrotic tissue, inflammation, and especially intraductal papilloma [46]. In fact, intraductal papilloma constitutes a high proportion in the BI‐RADS 4a category (≤ 10 mm vs. > 10 mm, 53% vs. 32%) (Figure S9), in which the proportion of malignancies (2% ~ 10%) is extremely low. As previously reported in the literature, the variable appearance of papillary lesions makes differentiation of benign from malignant pathologies difficult on imaging [47], and it is also difficult to gain a definite diagnosis by CNB, eventually resulting in overtreatment [48]. Moreover, for tumors with scattered cancer nests, CNB may miss the targeted point, causing a false‐negative result, even when the tumor size is larger than 2 cm [45]. Thus, it is necessary to develop a detection method to improve the accuracy of differentiating benign and malignant breast tumors, not only reducing the influence of man‐made factors but also enabling quicker diagnosis and keeping tumor integrity as much as possible. Liquid biopsy is gradually becoming a reality.

**FIGURE 5:**
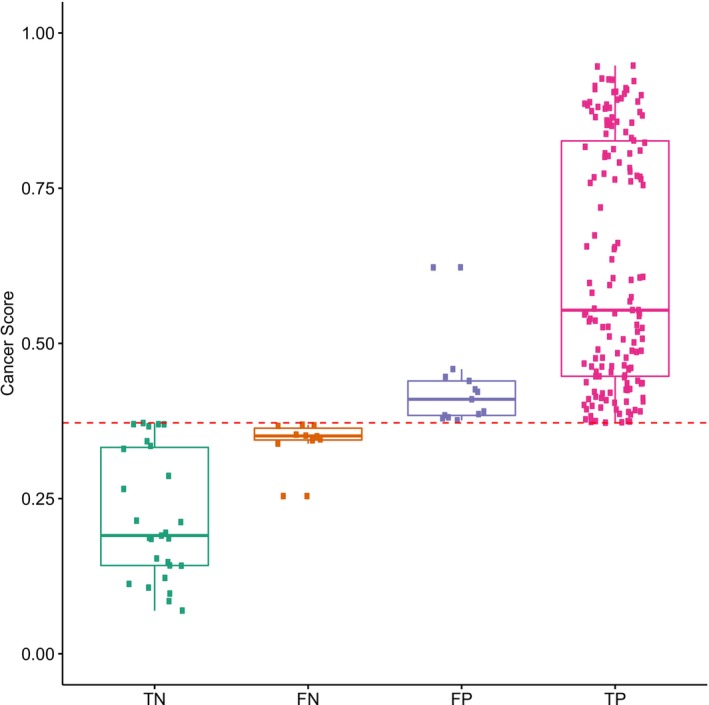
Distribution of the cancer scores among different types of the predicted outcomes by the methylation model. In a group of 204 patients where CNB pathology results matched postoperative pathology outcomes, the methylation model's TN (true‐negative predictions), FN (false‐negative predictions), FP (false‐positive predictions), and TP (true‐positive predictions) were calculated. *x*‐axis, the predicted cancer scores by the methylation model; The red line, the cutoff of the methylation model for diagnosing benign and malignant breast tumors.

In contrast to invasive core needle biopsy, the overall performance (Table S8) of diagnosing breast cancer by the current methylation model based on noninvasive liquid biopsy was slightly inferior. However, DNA methylation alterations occur prior to carcinogenesis [49]. For patients with false‐positive predictions by the methylation model, currently, due to the short follow‐up time, it cannot be completely considered a real false positive. Among patients with false‐negative predictions, 90% had cancer scores close to the threshold. The cancer scores of true‐negative and true‐positive predictions tended to be polarized (Figure 5). This result suggested that the methylation model was one that could specifically differentiate benign and malignant breast tumors. Increasing the frequency of follow‐up of these false‐positive or false‐negative patients may allow us to better monitor changes in progressive symptoms or evaluate the risk of postoperative recurrence or metastasis.

Due to the addition of more benign and precancerous lesions (atypical ductal hyperplasia and intraductal papilloma) as controls, our methylation panel has a good performance in improving the SE of early‐stage tumors in a noninvasive manner (stage 0 vs. stage I: 85.71% vs. 78.57%) and is the first to use targeted methylation sequencing to differentiate benign and malignant breast tumors thus far. The research by Liu et al. did not include precancerous lesions in the analysis, and the development of their panel is not rigorous enough with just a training set and a validation set (detection rate of stage I: 87.5%, 14/16) [12]. Previous large studies focusing on the detection of multiple cancers via methylation signatures in ctDNA were different from ours in terms of differentiating benign and malignant breast diseases. They involved efforts to compare the methylation patterns in serum between healthy people and cancer patients instead of taking benign patients as a control group. Because of the limitations of the research design in those studies, the sensitivity of stage I breast cancer was lower (< 10%–43%) [50, 51], likely affected by the enrolled sample type, disease stage, and cancer distribution.

In this study, we also clarified that the 103‐marker methylation model had the potential to discriminate DCIS from benign lesions, precancerous lesions, and IDC (*t* test, *p* < 0.05) (Figure 3). This finding suggests a promising avenue for improving detection rates where traditional methods such as ultrasonography or mammography fall short. However, due to the small number of DCIS patients enrolled, our results are preliminary. We could not obtain the cutoff value to distinguish DCIS from IDC at present.

Nevertheless, there were several limitations in our study. First, it was a single‐centre retrospective study, and the number of samples used to represent each stage was limited. We will validate the performance of the model at different sites to eliminate interference from other sites. Second, although it has been reported that ctDNA has the ability to be a promising prognostic and possibly predictive biomarker in patients with cancers [52], the independent test sets of our study did not involve complete follow‐up, by which the performance of the methylation model on prognosis needs to be further verified. Third, our current study is based on a retrospective analysis, and therefore, prospective studies are needed to validate the conclusions. We expect that methylation detection could help to reduce the false‐negative rate by CNB and the false‐positive rate by ultrasonography or mammography and even be used for early diagnosis, therapeutic monitoring, and follow‐up, independent of other examinations. Fourth, although our 103‐marker model demonstrated high sensitivity in detecting malignant tumors, its specificity for benign lesions was relatively limited. In both the validation and independent test cohorts, a substantial proportion of benign samples were misclassified as malignant. This underscores the need for further refinement of the model to enhance its discriminative performance between benign and malignant conditions. Future optimization should prioritize large‐scale prospective clinical trials to improve model robustness and achieve a more optimal balance between sensitivity and specificity.

## 4 Conclusion

In summary, the 103‐marker methylation model exhibited powerful performance in distinguishing breast cancer from benign tumors and high accuracy in early diagnosis and could compensate for imaging defects when combined with ultrasonography or mammography. In patients with tumors not suitable for core needle biopsy, methylation detection offers a high negative predictive value to avoid overtreatment.

## 5 Materials and Methods

### 5.1 Study Design and Participants

The study design is described in Figure S1. A breast cancer‐specific methylation panel consisting of 13,676 differentially methylated regions (DMRs) of breast cancer was developed according to the AnchorDx breast cancer‐specific methylation database and The Cancer Genome Atlas (TCGA) database. This panel covered 129,794 3‐CpG markers. A 3‐CpG marker is defined as a region with three sequential CpGs. Each DMR may include several 3‐CpG markers. One hundred and twelve paired breast tissue‐plasma samples (benign: malignant = 56:56) and 40 white blood cell samples (WBCs) (benign: malignant = 20:20) (Table S9) were applied for methylation marker screening to find reliable breast cancer‐specific methylation signals with a lower noise background. In addition, another 307 plasma samples (benign: malignant = 153:154) (Table S10) were used to identify plasma‐specific markers owing to the possibly different methylation patterns between genomic DNA (gDNA) and cfDNA. Finally, 2272 breast cancer‐specific methylated 3‐CpG biomarkers were chosen for model development.

The plasma samples collected from 504 (Table S5) female patients who underwent breast ultrasonography and breast tissue resection were assigned to four cohorts (excluding 3 failures): 214 were in the training set, 93 were in the validation set, and 88 and 106 were separately in two independent test sets (Table S10). Plasma samples from the training and validation sets were collected from 2017 to 2018, those from independent test set 1 (IndTes‐1) were collected from 2019 to 2020, and those from the other independent test set (IndTes‐2) were collected in 2021. Differences regarding molecular subtypes of malignant tumors were included for hormone receptor‐positive and human epidermal growth factor receptor 2‐negative HR+/HER2− breast cancer (luminal A and luminal B with HER2−), HER2+ breast cancer (luminal B with HER2+ and HER2+), and triple‐negative breast cancer (TNBC).

A 103‐marker methylation model was ultimately chosen from 307 plasma samples for diagnosing benign and malignant breast tumors. An independent tissue cohort (benign: malignant = 56:209, Table S11) was used to verify the methylation signals of the 103 markers in breast cancer. Two independent test sets were used to validate the performance of the methylation model. The performance of diagnosing breast cancer in the BI‐RADS 4 category was compared between the 103‐marker model and breast ultrasonography in the same patients in the combined independent test set.

### 5.2 Sample Collection

The clinical samples of this study, including blood and fresh frozen breast tissue samples, were obtained from the Department of Breast Surgery, Harbin Medical University Cancer Hospital. The study was approved by The Ethics Committee of Harbin Medical University Cancer Hospital. Written consent was collected from each participant.

After the patients were enrolled, 10 mL of peripheral blood was drawn into Cell‐Free DNA BCT blood collection tubes (Streck BCTs, Streck, Omaha NE. Cat# 218962) before surgery. Plasma and WBCs were isolated within 48 h after blood collection and immediately stored in a − 80°C freezer until use. Repeated freezing and thawing of plasma were avoided to prevent cfDNA degradation and genomic DNA contamination from WBCs. Breast lesion tissues were sampled at the time of surgery, snap‐frozen in liquid nitrogen, and stored at −80°C. All fresh frozen tissue samples underwent research histopathology review by an expert pathologist before DNA extraction.

Patient follow‐up is ongoing on a schedule. All patients will be followed regularly for at least 5 years or until death.

### 5.3 DNA Extraction

Genomic DNA (gDNA) was isolated from fresh frozen tissue samples and WBCs using the DNeasy Blood & Tissue Kit (Qiagen, Cat# 69506) according to the manufacturer's protocol. gDNA was quantified using the Qubit dsDNA HS Assay Kit (Thermo Fisher Scientific, Cat# Q32854, Eugene, Oregon, USA) and fragmented to 200 bp (peak size) by an M220 Focused‐ultrasonicator (Covaris Inc., Boston, Massachusetts, USA). Fragmented DNA was qualified by agarose gel electrophoresis.

Cell‐free DNA (cfDNA) was extracted from plasma using the MagMAX CellFree DNA Isolation Kit (Thermo Fisher, Cat# A29319) according to the manufacturer's protocol. The concentration of cfDNA was measured by the Qubit dsDNA HS Assay Kit, and the purity was examined using the Agilent High Sensitivity DNA Kit (Agilent, Cat# 5067–4626, CA, USA).

### 5.4 AnchorIRIS Library Preparation and Sequencing

Qualified DNA was bisulfite‐treated and purified by the EZ DNA Methylation‐Lightning Kit (Cat# D5031, Zymo Research) to convert unmethylated cytosines into uracils. The required input of library preparation for gDNA is 50 ng and that for cfDNA is greater than 3 ng. Library preparation was performed using the AnchorDx EpiVisio Methylation Library Prep Kit (AnchorDx Inc., Guangzhou, China, Cat# A0UX00019) and AnchorDx EpiVisio Indexing PCR Kit (AnchorDx Inc., Guangzhou, China, Cat# A2DX00025) [16, 53, 54]. The concentration of prehybridization libraries was determined using the Qubit dsDNA HS Assay Kit. Qualified prehybridization libraries should contain more than 400 ng DNA for target enrichment.

Target enrichment was performed using the AnchorDx EpiVisio Target Enrichment Kit (AnchorDx, catalog A0UX00031) [16] and an AnchorDx Breast Cancer‐specific methylation panel consisting of 13,676 preselected regions enriched for breast cancer‐specific methylation. The enriched libraries were sequenced on the NovaSeq 6000 System (Illumina Inc.). The pipeline for processing raw sequencing data and the criteria for evaluating sequencing quality were described in previous research [16].

### 5.5 Breast Cancer–Specific Methylation Panel Development

The breast cancer‐specific methylation panel was generated from the AnchorDx breast cancer‐specific methylation database and TCGA database, which included 13,676 differentially methylated regions in breast cancer consisting of 129,794 3‐CpG markers (Figures S1 and S2A,B).

The AnchorDx breast cancer‐specific methylation database was developed based on 338 breast tissue samples (benign: malignant = 55:283) that were merged into 71 methylated pools, including 11 benign pools, 24 HR+/HER2‐ pools (1 QC failed), 25 HER2+ pools, and 10 TNBC pools, and were analyzed by the TruSeq Methyl Capture EPIC Library Kit (Illumina, Cat # FC‐151‐1002).

The data preprocessing and analysis of differentially methylated CpG sites were described in previous studies [16]. There were two types of methylated CpG sites selected for AnchorDx breast cancer‐specific methylation panel design—common tumor markers and tumor subtype‐specific markers. The common tumor markers that differentiated all the compared groups (malignant vs. benign; TNBC vs. benign; HR+/HER2− vs. benign; HER2+ vs. benign) were screened by the following criteria: *p* < 0.001, area under the curve (AUC) > 0.9, mean beta value < 0.2 in the hypomethylated group and Δ (i.e., group difference) > 0.1 (Figure S2C). The tumor subtype‐specific markers identified only one of the compared groups (i.e., TNBC vs. HR+/HER2− and HER2+; HR+/HER2− vs. HER2+ and TNBC; HER2+ vs. HR+/HER2− and TNBC) by the following criteria: *p* < 0.001 and AUC > 0.8 (Figure S2D).

### 5.6 DNA Methylation Pattern Analysis

The data preprocessing from FASTQ files to methylation count files was described in previous studies [16]. DNA methylation patterns were described as contiguous 3‐CpG areas within 200 bp. The comethylated reads were defined as reads having all consecutive methylated CpGs within a sliding window of three consecutive CpGs, and the methylation level referred to the percentage of comethylated reads: comethylated reads/all mapped reads with three consecutive CpGs. The 3‐CpG methylation patterns were used to create a predictive model for the diagnosis of benign and malignant breast lesions.

### 5.7 Benign‐Malignant Prediction Modeling

The methylation level of the methylation patterns was calculated in the cohorts of 112 paired tissue‐plasma samples, 40 WBC samples (Figure S3) and 307 plasma samples, including 214 samples in the training set and 93 samples in the validation set. The filtering conditions were as follows: false discovery rate (FDR) < 0.01 and Δ > 0.05 in the compared groups (i.e., 56 tissue malignant samples vs. 56 tissue benign samples; 56 tissue malignant samples vs. 40 WBCs), *p* < 0.05 in the compared groups (i.e., 56 paired plasma malignant samples vs. 56 paired plasma benign samples), and *p* < 0.01 in the compared groups (i.e., 107 training plasma malignant samples vs. 107 training plasma benign samples). Next, random forest (RF) and least absolute shrinkage and selection operator (LASSO) models in the training set were used to narrow down the number of markers. Finally, a total of 103 markers were identified. Gene set annotation and functional enrichment of 103 markers were implemented by the Metascape website (http://metascape.org/gp/index.html).

Using the random forest method, we constructed a diagnostic prediction model by the 103 selected markers that preferentially discriminated malignant samples from benign samples in the independent test datasets (Figure 1B).

### 5.8 Combined Model With Methylation Markers and Ultrasonography BI‐RADS

The class of ultrasonography Breast Imaging Reporting and Data System (BI‐RADS) (i.e., 1, 2, 3, 4a, 4b, 4c, 5, and 6) was numerical (i.e., 1, 2, 3, 4.3, 4.5, 4.7, 5, and 6). A logistic regression model of ultrasonography BI‐RADS was used to calculate the malignancy probability as a function of 1 variable:

$$\text{probability of malignancy}\;\left(\mathrm{POM}\right)=\frac{1}{\left(1+e^{-x}\right)},$$

$$x=-11.9557+\left(2.7311\times \mathrm{BI}-{\text{RADS}}_{\text{ultrasound}}\right),$$

where *e* is Euler's number, a mathematical constant approximately equal to 2.71828. Subsequently, a logistic regression model combining 103 markers with ultrasonography BI‐RADS was also built in the validation set to calculate the malignancy probability as a function of 2 variables:

$$\mathrm{POM}=\frac{1}{\left(1+e^{-x}\right)}$$

$$\begin{array}{c} x=-8.785+\left(10.217\times {\mathrm{POM}}_{103-\text{marker random forest model}}\;\right)\\ +\left(7.720\times {\mathrm{POM}}_{\text{ultrasound}\;\mathrm{BI}-\text{RADS logistic regression model}}\right), \end{array}$$

where *e* = 2.71828.

### 5.9 Combined Model With Methylation Markers and Mammography BI‐RADS

The class of mammography Breast Imaging Reporting and Data System (BI‐RADS) (i.e., 1, 2, 3, 4a, 4b, 4c, 5, and 6) was numerical (i.e., 1, 2, 3, 4.3, 4.5, 4.7, 5, and 6). A logistic regression model of mammography BI‐RADS was used to calculate the malignancy probability as a function of 1 variable:

$$\text{probability of malignancy}\;\left(\mathrm{POM}\right)=\frac{1}{\left(1+e^{-x}\right)},$$

$$x=-3.2584+\left(0.8649\times \mathrm{BI}-{\text{RADS}}_{\text{mammogram}}\right),$$

where *e* is Euler's number, a mathematical constant approximately equal to 2.71828. Subsequently, a logistic regression model combining 103 markers with mammography BI‐RADS was also built in the validation set to calculate the malignancy probability as a function of 2 variables:

$$\mathrm{POM}=\frac{1}{\left(1+e^{-x}\right)}$$

$$\begin{array}{c} x=-6.462+\left(9.328\times {\mathrm{POM}}_{103-\text{marker random forest model}}\;\right)\\ +\left(3.842\times {\mathrm{POM}}_{\text{mammogram}\;\mathrm{BI}-\text{RADS logistic regression model}}\right), \end{array}$$

where *e* = 2.71828.

### 5.10 Statistics

For marker selection, the *p* value was calculated by the Mann–Whitney *U* test between the malignant and benign/WBC groups, and FDR (Benjamini–Hochberg method) correction was used for multiple test correction. Unless otherwise specified, all statistical tests were two‐tailed. The cancer score per sample was determined by the probability of the random forest machine leaning algorithm constructed from the training set using the 103‐marker methylation data, called the 103‐marker model. The diagnostic sensitivity, specificity, accuracy, PPV, and NPV of the 103‐marker model and combined models were calculated in both the validation and test sets. Positive and negative classifications of the 103‐marker model were determined by the cutoff value (0.372) under 95% sensitivity. Meanwhile, the combined model with methylation markers and ultrasonography or mammography BI‐RADS was determined by the cutoff value (combined ultrasonography: 0.185; combined mammography: 0.235) under 95% sensitivity. ROC curves were generated using the pROC R package (version 1.15.3). Kaplan–Meier curves for DFS were calculated and plotted with the “survival” (version 3.2.11) and “survminer” (version 0.4.9) R packages, and cancer score (model probability) cutoffs were determined by the SROC Youden cutoff (plasma: 0.83; tissue: 0.9905) with the “survivalROC” R package (version 1.0.3). All statistical analyses were performed with R software, version 4.0.0.

## Author Contributions

**Xianyu Zhang:** conceptualization (equal), project administration (equal), writing – original draft (lead), writing – review and editing (lead). **Yanling Yin:** project administration (equal), writing – original draft (equal), writing – review and editing (equal). **Zhujia Ye:** data curation (equal), writing – original draft (equal). **Xingda Zhang:** project administration (equal), writing – original draft (equal). **Wei Wei:** data curation (equal), writing – original draft (equal). **Yi Hao:** data curation (equal), writing – review and editing (equal). **Liuhong Zeng:** data curation (equal), methodology (equal), visualization (equal), writing – review and editing (equal). **Ting Yang:** formal analysis (equal), methodology (equal). **Dalin Li:** resources (equal). **Jun Wang:** data curation (equal), writing – review and editing (equal). **Dezhi Zhao:** formal analysis (equal), writing – review and editing (equal). **Yanbo Chen:** resources (equal). **Shan Lei:** data curation (equal), methodology (equal). **Yongdong Jiang:** resources (equal). **Youxue Zhang:** resources (equal). **Shouping Xu:** resources (equal). **Abiyasi Nanding:** formal analysis (equal), writing – review and editing (equal). **Yajie Gong:** formal analysis (equal). **Siwei Li:** formal analysis (equal). **Yuanyuan Yu:** data curation (equal). **Shilu Zhao:** formal analysis (equal). **Siyu Liu:** formal analysis (equal). **Yashuang Zhao:** formal analysis (equal), supervision (equal), writing – review and editing (equal). **Zhiwei Chen:** methodology (equal), supervision (equal), validation (equal). **Shihui Yu:** formal analysis (equal), writing – review and editing (equal). **Jian‐Bing Fan:** conceptualization (equal), data curation (equal), supervision (equal), writing – review and editing (equal). **Da Pang:** conceptualization (equal), project administration (equal), supervision (equal), writing – review and editing (equal).

## Ethics Statement

The study was approved by The Ethics Committee of Harbin Medical University Cancer Hospital.

## Consent

All participants provided written informed consent prior to inclusion in the study.

## Conflicts of Interest

Eight authors are employees of AnchorDx Medical Co. Ltd. (Guangzhou, China). This employment did not influence the study design, data analysis, interpretation, or conclusions. The remaining authors declare no conflicts of interest related to this research article.

## Supporting information


Data S1.

## Data Availability

The public datasets that support the findings of this study are available in The Cancer Genome Atlas (TCGA) database. All other data supporting the findings of this study are available from the corresponding author on reasonable request.
