# Supplementary material for: An Approach for Differential Diagnosis of Breast Tumors by ctDNA Methylation Sequencing
Source: Cancer Med. 2025 Jun 20;14(12):e71004. doi: 10.1002/cam4.71004 (PMC12180084; doi:10.1002/cam4.71004)
Supplement: Supplementary file 1 — Data S1. [file CAM4-14-e71004-s001.zip › cam471004-sup-0001-FigureS1.docx]

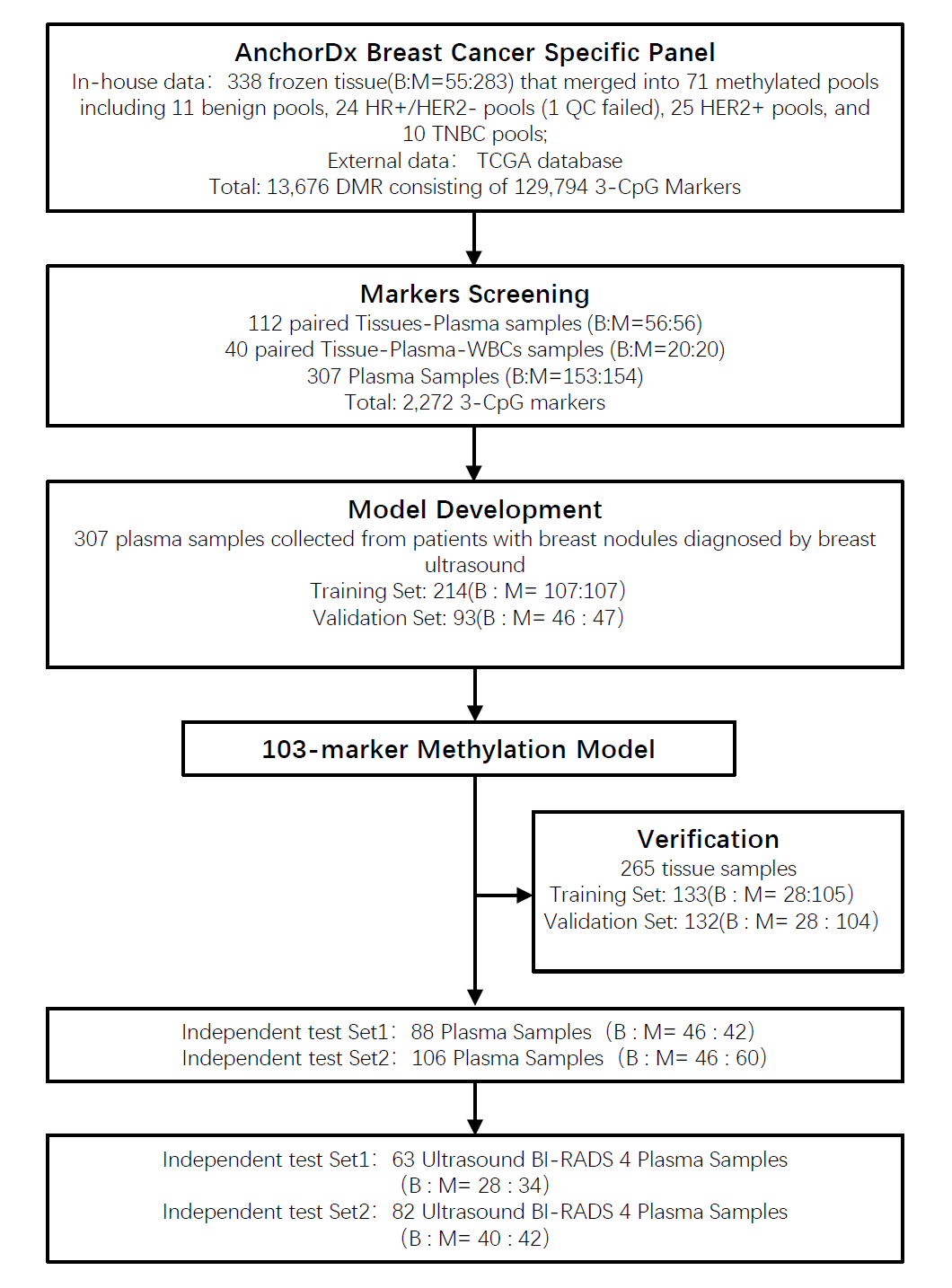


**Figure S1. Workflow of the study design.**

A total of 13,676 differentially methylated regions (DMRs) of breast cancer consisting of 129,794 3-CpG markers were identified based on the AnchorDx breast cancer-specific methylation database and TCGA database. A total of 112 paired breast tissue-plasma samples, 40 white blood cell samples, and 307 plasma samples were used to identify a total of 2,272 methylated 3-CpG markers. Patients with breast tumors (307 plasma samples and 265 tissue samples) were used for model development. The final performance of our model was tested in two independent test cohorts containing 88 (B:M=46:42) and 106 (B:M=46:60) plasma samples from patients with breast diseases. BI-RADS, Breast Imaging-Reporting and Data System.


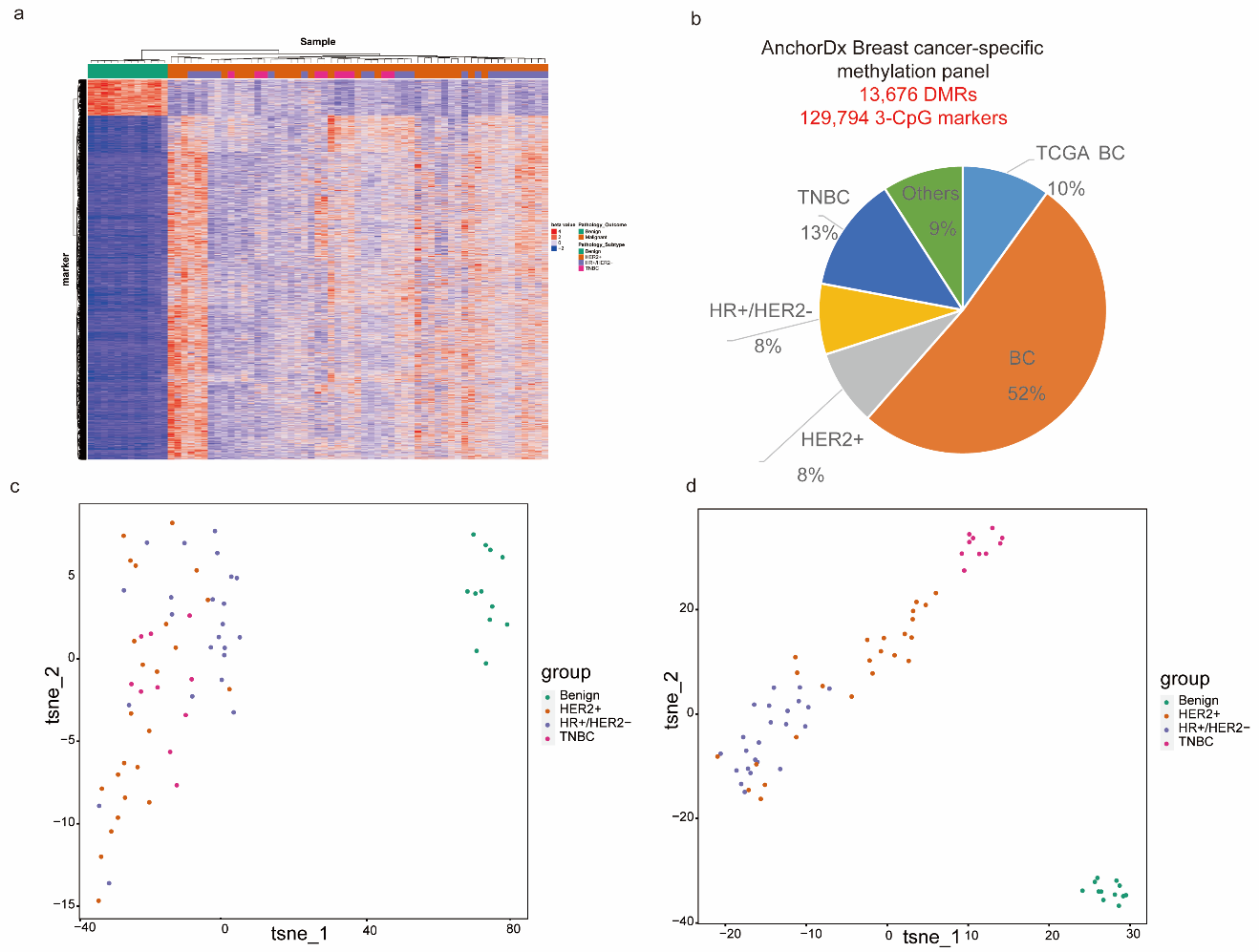


**Figure S2. Development of a breast cancer-specific methylation panel.** (a) Unsupervised hierarchical cluster heatmap of 129,794 3-CpG methylation markers. These markers were developed from 338 breast tissue samples (benign: malignant = 55: 283; these tissue samples were merged into 11 benign pools, 24 HR+/HER2- pools [1 QC failed], 25 HER2+ pools, and 10 TNBC pools) and the TCGA database. (b) Compositions of the AnchorDx breast cancer-specific methylation panel. Principal component analysis of the common tumor markers (c) and the tumor subtype-specific markers (d) among different subtypes of breast lesions. ER, oestrogen receptor; PR, progesterone receptor; HER2, human epidermal growth factor receptor 2; TNBC, triple-negative breast cancer; TCGA, The Cancer Genome Atlas.


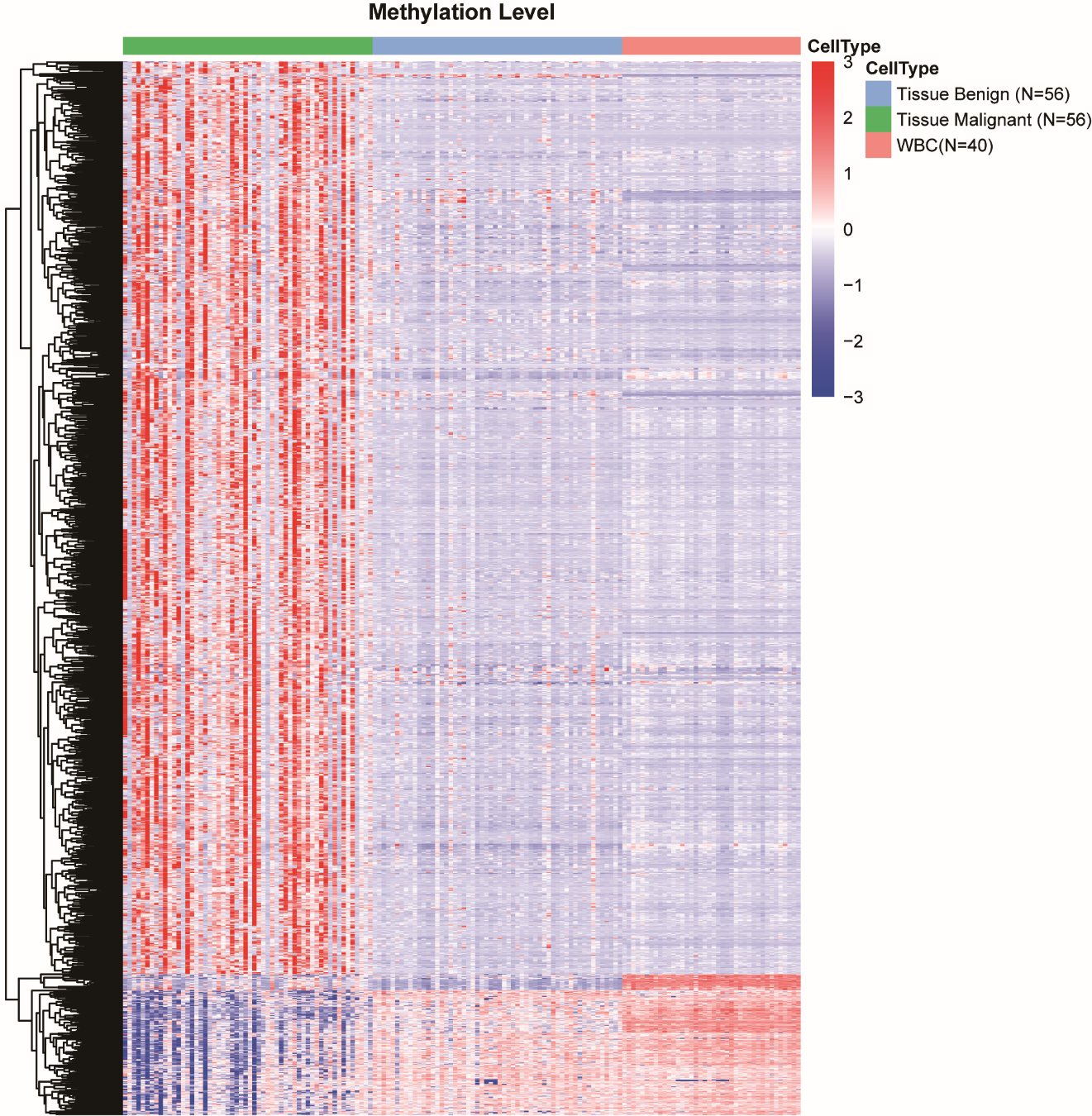


**Figure S3. Methylation signals of selected markers in tissue and white blood cell samples.**

Unsupervised hierarchical cluster heatmap of 2,272 3-CpG methylation markers differentially methylated in 112 tissue samples (benign: malignant = 56: 56) and 40 white blood cells.


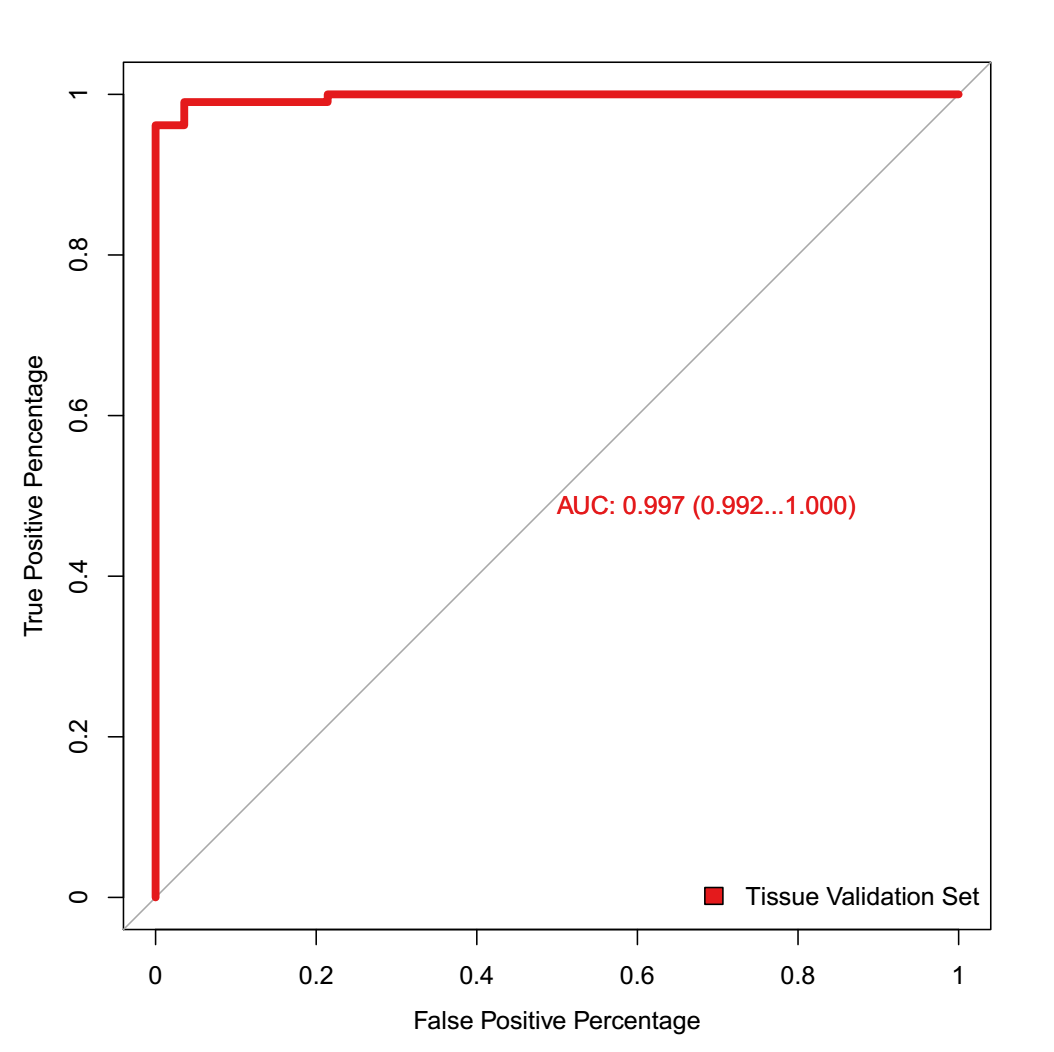


**Figure S4.** **Performance of the methylation model in the validation set of tissue samples.**

Representative receiver operating characteristic (ROC) curves of the 103-marker methylation model for differentiating benign and malignant breast nodules in the validation set of tissue samples. Benign (n=28), malignant (n=104).


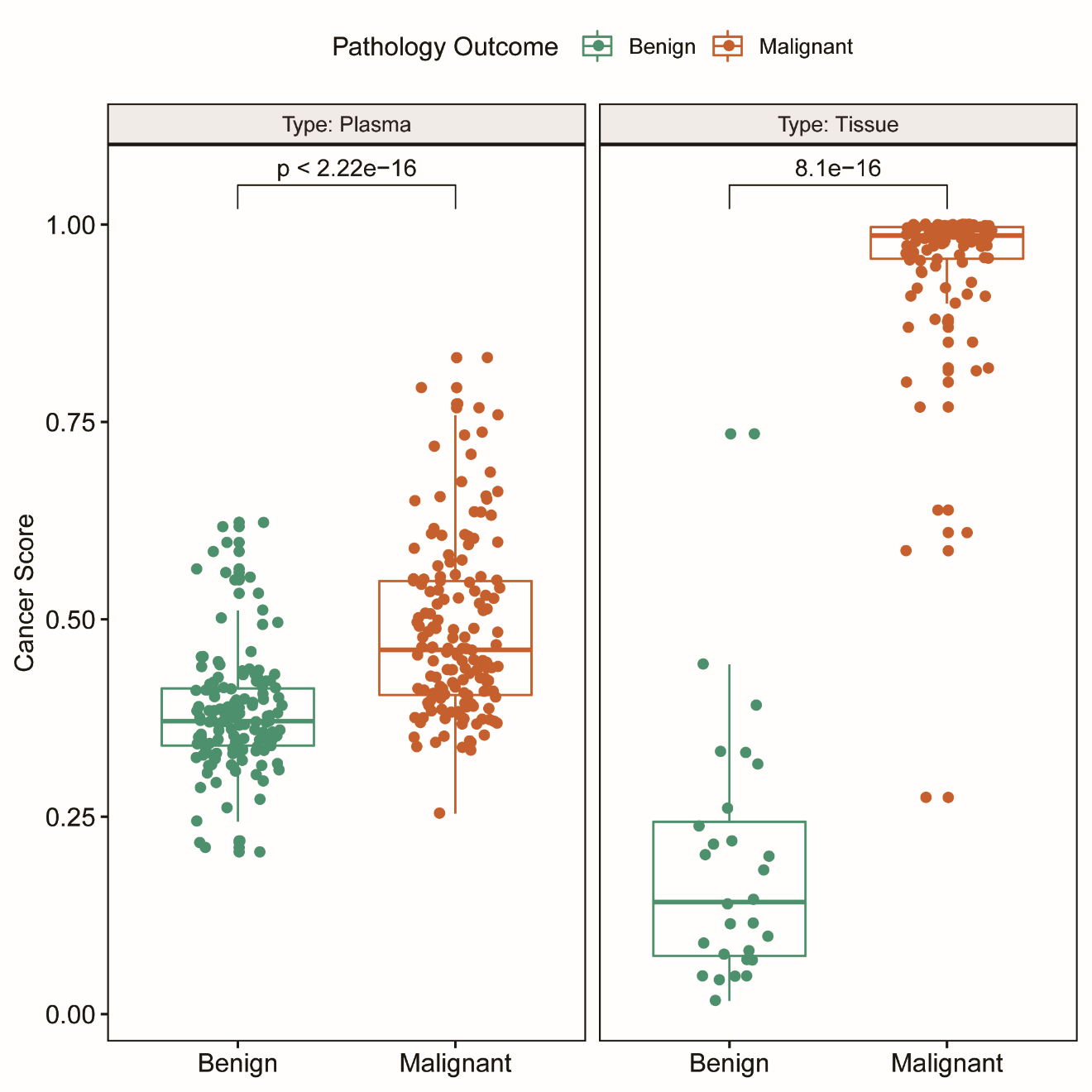


**Figure S5. Differences in methylation signals between benign and malignant breast nodules in plasma and tissue samples.**

Y-axis, the cancer scores predicted by the 103-marker methylation model. The Wilcoxon test was used to determine statistical significance between groups based on cancer scores. Statistical significance is indicated by a p-value less than 0.05.


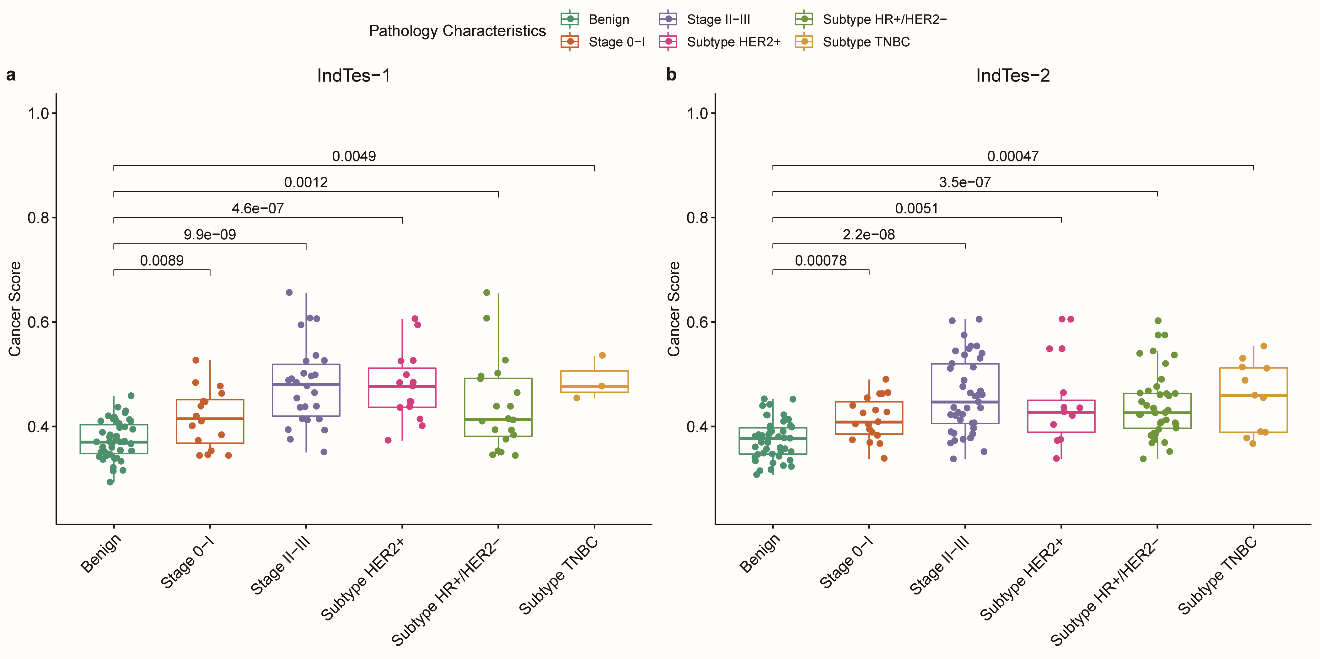


**Figure S6. Performance of the 103-marker model in different groups of the two independent test sets.**

DNA methylation signals of the 103-marker model in (a) independent test set 1 (IndTes-1) and (b) independent test set 2 (IndTes-2) in different groups: benign (IndTes-1: n=46; IndTes-2: n=46), stages 0-I (IndTes-1: n=16; IndTes-2: n=19), stages II-III (IndTes-1: n=26; IndTes-2: n=41), TNBC (IndTes-1: n=3; IndTes-2: n=11), (IndTes-1: n=20; IndTes-2: n=37), and HER2+ (IndTes-1: n=15; IndTes-2: n=11). The Wilcoxon test was used to determine statistical significance between groups based on cancer scores. Statistical significance is indicated by a p-value less than 0.05.


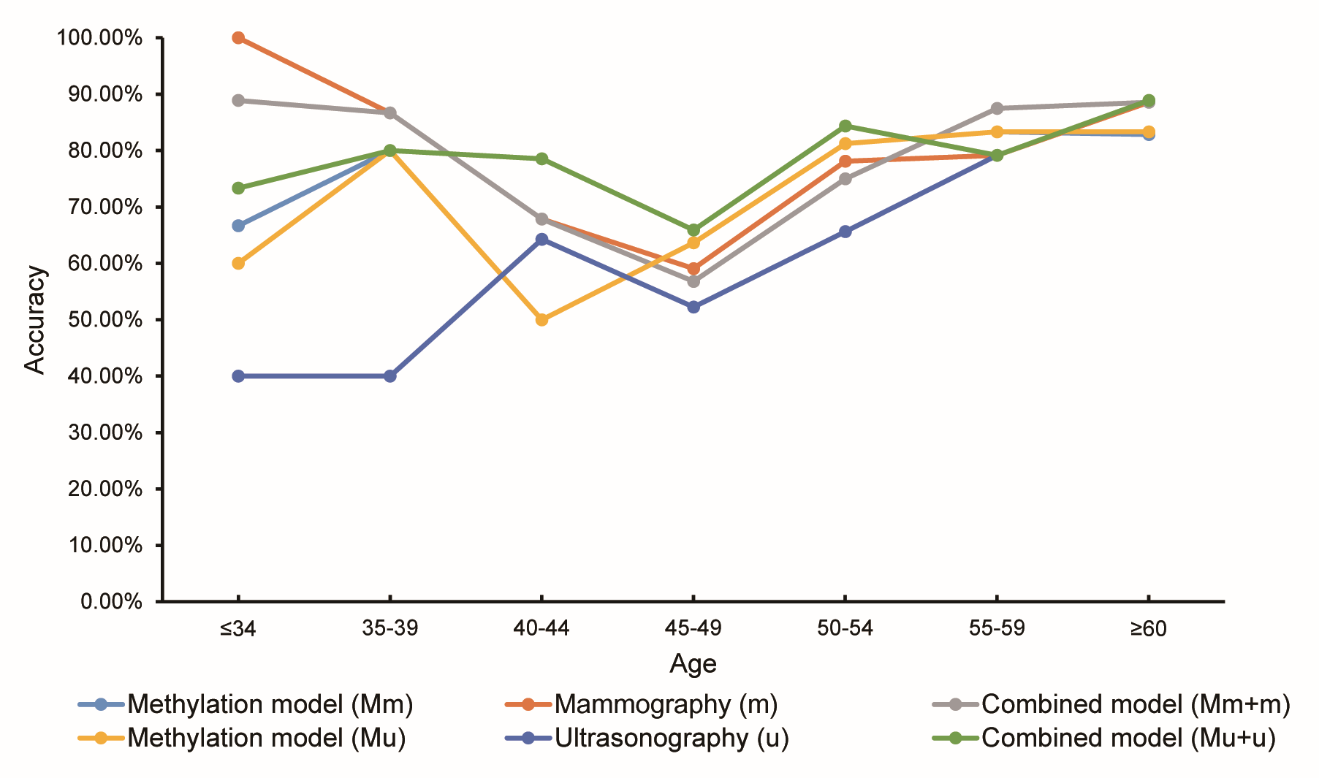


**Figure S7. Comparison of the diagnostic performance in different age groups.**

Comparison of the diagnostic performance of breast ultrasonography, mammography, the methylation model, and the combined model in different age groups (less than 34, 35-39, 40-44, 45-49, 50-54, 55-59, more than 60) in the combined independent test sets. Mm: methylation model in the mammography group; m: mammography; Mm+m: combined model of methylation and mammography; Mu: methylation model in the ultrasonography group; u: ultrasonography; Mu+u: combined model of methylation and ultrasonography.


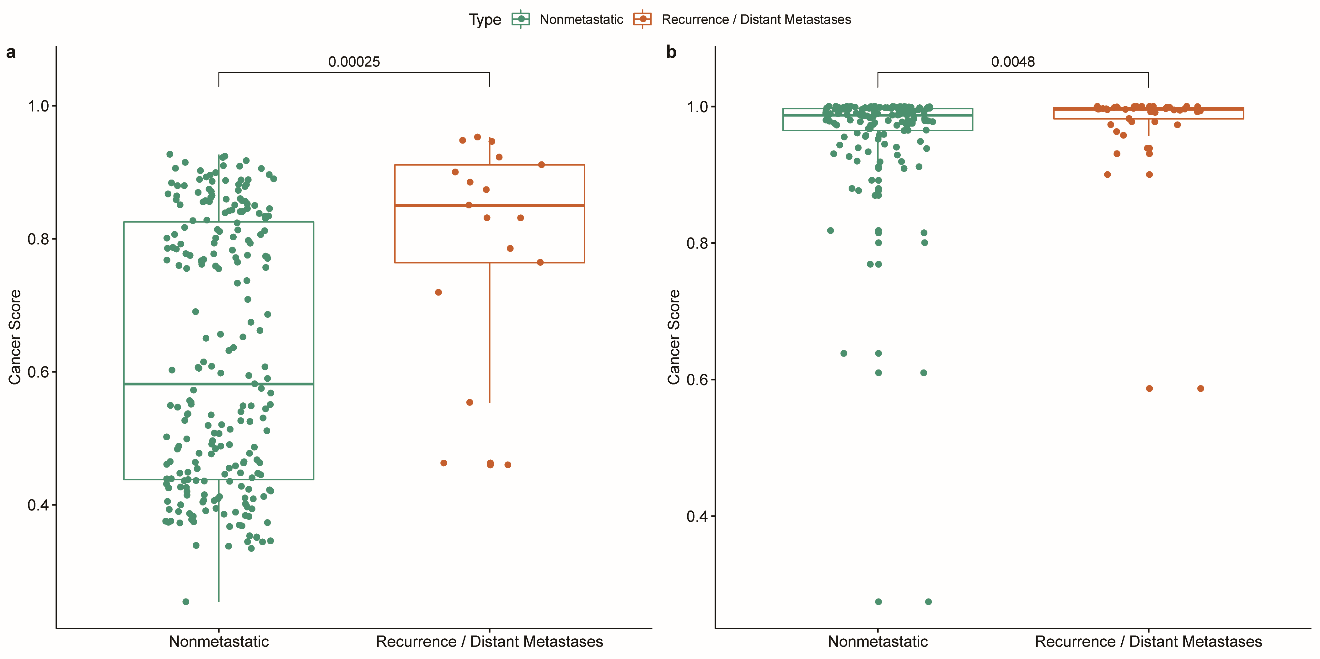


**Figure S8. Differences in methylation signals between nonmetastatic and metastatic breast cancers.**

Y-axis, the cancer scores predicted by the 103-marker methylation model in (a) 252 plasma samples and (b) 182 tissue samples. The Wilcoxon test was used to determine statistical significance between groups based on cancer scores. Statistical significance is indicated by a p-value less than 0.05.


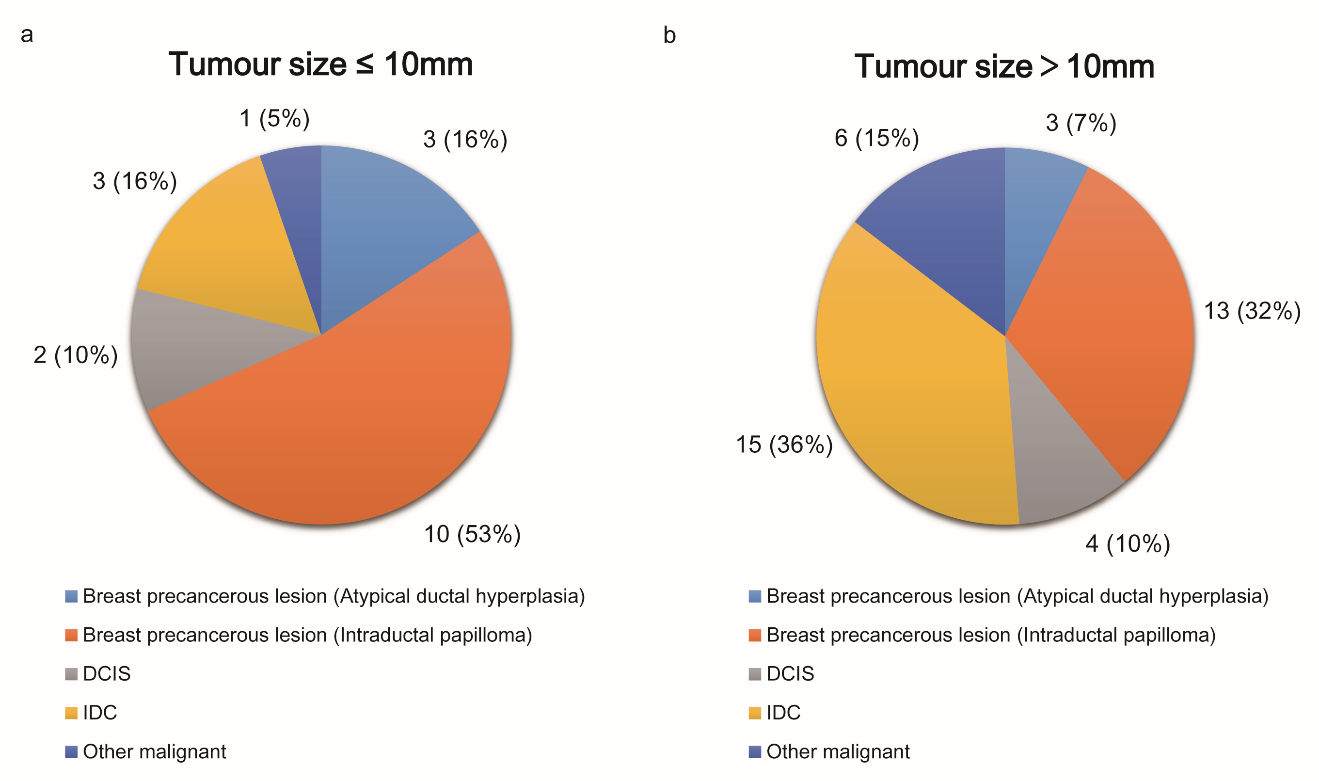


**Figure S9. Distribution of the different pathological types of breast lesions in BI-RADS 4a patients.**

(a) Tumor size ≤ 10 mm. (b) Tumor size > 10 mm.
