# Supplementary material for: An Approach for Differential Diagnosis of Breast Tumors by ctDNA Methylation Sequencing
Source: Cancer Med. 2025 Jun 20;14(12):e71004. doi: 10.1002/cam4.71004 (PMC12180084; doi:10.1002/cam4.71004)
Supplement: Supplementary file 1 — Data S1. [file CAM4-14-e71004-s001.zip › cam471004-sup-0003-TableS4.docx]

**Table S1. Functional enrichment of selected methylation markers.**

[Excel file]

**Table S2. Gene annotation of selected methylation markers.**

[Excel file]

**Table S3. Comparison of the performance in the diagnosis of breast cancer between the methylation model, breast ultrasonography, mammography and combined models.**

| **Cohort** | **Detection** | **Ultrasonography** | **Methylation model** | **Combined model U+M** | **Mammography** | **Methylation model** | **Combined model M+M** |
| --- | --- | --- | --- | --- | --- | --- | --- |
| Validation set | SE | 95.74% (45/47) | 95.74% (45/47) | 95.74% (45/47) | 84.09% (36/43) | 97.73% (43/44) | 95.45% (42/44) |
| Benign (n=46) | SP | 32.61% (15/46) | 47.83% (22/46) | 63.04% (29/46) | 68.89% (31/45) | 46.67% (21/45) | 55.56% (25/45) |
| Malignant (n=47) | ACC | 64.52% (60/93) | 72.04% (67/93) | 79.57% (74/93) | 76.40% (68/89) | 71.91% (64/89) | 75.28% (67/89) |
| Independent test | SE | 99.02% (101/102) | 89.22% (91/102) | 96.08% (98/102) | 89.11% (90/101) | 89.11% (90/101) | 92.08% (93/101) |
| set-1+2 | SP | 26.09% (24/92) | 52.17% (48/92) | 58.70% (54/92) | 60.47% (52/86) | 52.33% (45/86) | 55.81% (48/86) |
|  | ACC | 64.43% (125/194) | 71.65% (139/194) | 78.35% (152/194) | 75.94% (142/187) | 72.19% (135/187) | 75.40% (141/187) |

Abbreviations: SE, Sensitivity; SP, Specificity; ACC, Accuracy; U+M, Ultrasonography+ Methylation; M+M, Mammography+ Methylation.

**Table S4. Comparison of the performance in the diagnosis of BI-RADS 4a breast cancer between the methylation, breast ultrasonography, mammography and combined models.**

| **Cohort** | **Detection** | **Ultrasonography** | **Methylation model** | **Combined model (U+M)** | **Mammography** | **Methylation model** | **Combined model (M+M)** |
| --- | --- | --- | --- | --- | --- | --- | --- |
| Validation set | ACC | 21.21% (7/33) | 57.58% (19/33) | 57.58% (19/33) | 35.29% (6/17) | 41.18% (7/17) | 35.29% (6/17) |
| Independent test sets | ACC | 17.39% (12/69) | 57.97% (40/69) | 56.52% (39/69) | 43.14% (22/51) | 68.63% (35/51) | 43.14% (22/51) |

**Table S5. Basic information of breast mass patients who provided tissue samples and plasma samples for the study.**

[Excel file]

**Table S6. Prognostic factors in the Cox proportional hazards model (plasma).**

| Variables | Univariate analysis | | |  | Multivariate analysis | | |
| --- | --- | --- | --- | --- | --- | --- | --- |
|  | HR | 95%CI | *p*-value |  | HR | 95%CI | *p*-value |
| Cancer Score (< 0.83 vs. ≥ 0.83 ) | 0.21 | 0.08 - 0.57 | 0.0022 |  | 0.18 | 0.06 - 0.58 | 0.0041 |
| Age (< 50 vs. ≥ 50) | 1.92 | 0.68 - 5.46 | 0.2198 |  | 2.33 | 0.72 - 7.53 | 0.1585 |
| Pathology (None-DCIS vs. DCIS) | 0 | 0 - Inf | 0.9969 |  | 0 | 0 - Inf | 0.9975 |
| TNM stage (0-II vs. III) | 3.86 | 1.43 - 10.45 | 0.0078 |  | 2.9 | 0.84 - 9.97 | 0.0911 |
| Tumor Size (> 2 cm vs. ≤ 2 cm) | 2.49 | 0.88 - 7.07 | 0.0871 |  | 4.13 | 1.3 - 13.13 | 0.0162 |
| Histological Grade (0-II vs. III) | 0.52 | 0.18 - 1.48 | 0.2195 |  | 0.42 | 0.1 - 1.82 | 0.2465 |
| LNM (negative vs. positive) | 0.75 | 0.26 - 2.13 | 0.5919 |  | 0.48 | 0.13 - 1.82 | 0.2797 |
| ER status (negative vs. positive) | 0.19 | 0.07 - 0.5 | 8.00E-04 |  | 0.12 | 0.01 - 1 | 0.0501 |
| PR status (negative vs. positive) | 0.34 | 0.13 - 0.93 | 0.0354 |  | 2.1 | 0.25 - 17.74 | 0.497 |
| HER2 status (negative vs. positive) | 0.98 | 0.32 - 3 | 0.9671 |  | 0.38 | 0.1 - 1.43 | 0.1522 |
| KI67 status (negative vs. positive) | 1.94 | 0.68 - 5.5 | 0.2144 |  | 2.82 | 0.71 - 11.29 | 0.1423 |

**Table S7. Prognostic factors in the Cox proportional hazards model (tissue).**

| Variables | Univariate analysis | | |  | Multivariate analysis | | |
| --- | --- | --- | --- | --- | --- | --- | --- |
|  | HR | 95%CI | *p*-value |  | HR | 95%CI | *p*-value |
| Cancer Score (< 0.9905 vs. ≥ 0.9905 ) | 0.3 | 0.15 - 0.6 | 7.00E-04 | 0.37 | 0.17 - 0.84 | 0.0165 | 0.3 |
| Age (< 50 vs. ≥ 50) | 1.57 | 0.83 - 2.96 | 0.167 | 1.47 | 0.76 - 2.85 | 0.2527 | 1.57 |
| Pathology (None-DCIS vs. DCIS) | 0 | 0 - Inf | 0.9965 | 0 | 0 - Inf | 0.997 | 0 |
| TNM stage (0-II vs. III) | 2.5 | 1.35 - 4.63 | 0.0034 | 1.93 | 0.79 - 4.69 | 0.1466 | 2.5 |
| Tumor Size (> 2 cm vs. ≤ 2 cm) | 1.89 | 0.97 - 3.71 | 0.0633 | 1.57 | 0.77 - 3.22 | 0.2176 | 1.89 |
| Histological Grade (0-II vs. III) | 1.64 | 0.4 - 6.82 | 0.4945 | 1.69 | 0.37 - 7.62 | 0.4976 | 1.64 |
| LNM (negative vs. positive) | 2.29 | 1.21 - 4.33 | 0.0107 | 1.22 | 0.48 - 3.1 | 0.67 | 2.29 |
| ER status (negative vs. positive) | 0.62 | 0.33 - 1.15 | 0.128 | 0.63 | 0.28 - 1.46 | 0.2864 | 0.62 |
| PR status (negative vs. positive) | 0.51 | 0.26 - 0.98 | 0.0422 | 0.86 | 0.34 - 2.15 | 0.7431 | 0.51 |
| HER2 status (negative vs. positive) | 2.01 | 1.08 - 3.73 | 0.0267 | 1.16 | 0.55 - 2.46 | 0.6975 | 2.01 |
| KI67 status (negative vs. positive) | 1.06 | 0.54 - 2.07 | 0.8711 | 0.78 | 0.37 - 1.63 | 0.511 | 1.06 |

**Table S8. Comparison of the performance in the diagnosis of breast cancer between the methylation model and core needle biopsy.**

| Detection | Methylation model | Core needle biopsy |
| --- | --- | --- |
| SE | 94.15% (161/171) | 95.91% (164/171) |
| SP | 67.50% (27/40) | 100.00% (40/40) |
| ACC | 89.10% (188/211) | 96.68% (204/211) |

**Table S9. Characteristics of the paired tissue-plasma-WBC samples.**

|  | Paired tissue-plasma samples | | |  | Paired WBC samples | | |
| --- | --- | --- | --- | --- | --- | --- | --- |
|  | Benign (n=56) | Malignant (n=56) | Total  (n=112) |  | Benign (n=20) | Malignant (n=20) | Total  (n=40) |
| Age | | | | | | | |
| 40-49 | 44(78.6%) | 27(48.2%) | 71(63.4%) |  | 17(85.0%) | 10(50.0%) | 27(67.5%) |
| 50-59 | 12(21.4%) | 26(46.4%) | 38(33.9%) |  | 3(15.0%) | 7(35.0%) | 10(25.0%) |
| ≥60 |  | 3(5.4%) | 3(2.7%) |  |  | 3(15.0%) | 3(7.5%) |
| ultrasonography BI-RADS | | | | | | | |
| 1 | 1(1.8%) |  | 1(0.9%) |  |  |  |  |
| 3 | 24(42.9%) |  | 24(21.4%) |  | 9(45.0%) |  | 9(22.5%) |
| 4 | 31(55.4%) | 41(73.2%) | 72(64.3%) |  | 11(55.0%) | 11(55.0%) | 22(55.0%) |
| 4a | 21(37.5%) | 6(10.7%) | 27(24.1%) |  | 6(30.0%) | 3(15.0%) | 9(22.5%) |
| 4b | 8(14.3%) | 13(23.2%) | 21(18.8%) |  | 3(15.0%) | 2(10.0%) | 5(12.5%) |
| 4c | 2(3.6%) | 22(39.3%) | 24(21.4%) |  | 2(10.0%) | 6(30.0%) | 8(20.0%) |
| 5 |  | 13(23.2%) | 13(11.6%) |  |  | 8(40.0%) | 8(20.0%) |
| 6 |  | 2(3.6%) | 2(1.8%) |  |  | 1(5.0%) | 1(2.5%) |
| mammography BI-RADS |  |  |  |  |  |  |  |
| 1 | 9(16.1%) | 2(3.6%) | 11(9.8%) |  | 3(15.0%) |  | 3(7.5%) |
| 2 | 9(16.1%) | 2(3.6%) | 11(9.8%) |  | 2(10.0%) | 1(5.0%) | 3(7.5%) |
| 3 | 16(28.6%) | 2(3.6%) | 18(16.1%) |  | 7(35.0%) | 1(5.0%) | 8(20.0%) |
| 4 | 22(39.3%) | 37(66.1%) | 59(52.7%) |  | 8(40.0%) | 12(60.0%) | 20(50.0%) |
| 4a | 17(30.4%) | 4(7.1%) | 21(18.8%) |  | 6(30.0%) | 1(5.0%) | 7(17.5%) |
| 4b | 4(7.1%) | 20(35.7%) | 24(21.4%) |  | 1(5.0%) | 5(25.0%) | 6(15.0%) |
| 4c | 1(1.8%) | 12(21.4%) | 13(11.6%) |  | 1(5.0%) | 6(30.0%) | 7(17.5%) |
| 4 without subcategory |  | 1(1.8%) | 1(0.9%) |  |  |  | (0.0%) |
| 5 |  | 9(16.1%) | 9(8.0%) |  |  | 3(15.0%) | 3(7.5%) |
| 6 |  | 3(5.4%) | 3(2.7%) |  |  | 2(10.0%) | 2(5.0%) |
| Unknown |  | 1(1.8%) | 1(0.9%) |  |  | 1(5.0%) | 1(2.5%) |
| Stage |  |  |  |  |  |  |  |
| 0 |  | 2(3.6%) | 2(1.8%) |  |  |  |  |
| I |  | 19(33.9%) | 19(17.0%) |  |  | 4(20.0%) | 4(10.0%) |
| II |  | 25(44.6%) | 25(22.3%) |  |  | 10(50.0%) | 10(25.0%) |
| III |  | 10(17.9%) | 10(8.9%) |  |  | 6(30.0%) | 6(15.0%) |
| Molecular Subtypes |  |  |  |  |  |  |  |
| HER2+ |  | 17(30.4%) | 17(15.2%) |  |  | 7(35.0%) | 7(17.5%) |
| HR+/HER2- |  | 33(58.9%) | 33(29.5%) |  |  | 11(55.0%) | 11(27.5%) |
| TNBC |  | 6(10.7%) | 6(5.4%) |  |  | 2(10.0%) | 2(5.0%) |

**Table S10: Characteristics of the patients in the training, validation and two independent test sets**

|  | **Training set** | | | | | | |  | **Validation set** | | | | | | | | |  | **Independent test set-1** | | | |  | **Independent test set-2** | | |  |  |
| --- | --- | --- | --- | --- | --- | --- | --- | --- | --- | --- | --- | --- | --- | --- | --- | --- | --- | --- | --- | --- | --- | --- | --- | --- | --- | --- | --- | --- |
|  | **Benign (n=107)** | | | **Malignant (n=107)** | | | |  | **Benign (n=46)** | | | | | **Malignant (n=47)** | | | |  | **Benign (n=46)** | | | **Malignant (n=42)** |  | **Benign**  **(n=46)** | | **Malignant (n=60)** |  |  |
| Age | |  | | | | |  | | | | | | | |  | | | | | | | |  |  |  |  |  |  |
| ＜40 | 5(4.7) | | | 2(1.9) | | | |  | 2(4.3) | | | | | 1(2.1) | | | |  | 8(17.4) | | |  |  | 16(34.8) | | 6(10.0) |  |  |
| 40-49 | 68(63.6) | | | 48(44.9) | | | |  | 34(73.9) | | | | | 25(53.2) | | | |  | 22(47.8) | | | 12(28.6) |  | 21(45.7) | | 17(28.3) |  |  |
| 50-59 | 34(31.8) | | | 52(48.6) | | | |  | 9(19.6) | | | | | 14(29.8) | | | |  | 14(30.4) | | | 17(40.5) |  | 5(10.9) | | 20(33.3) |  |  |
| ≥60 |  | | | 5(4.7) | | | |  | 1(2.2) | | | | | 7(14.9) | | | |  | 2(4.3) | | | 13(31.0) |  | 4(8.7) | | 17(28.3) |  |  |
| Ultrasonography  BI-RADS |  | | |  | |  | | | | |  | |  | | | |  | | | |  | |  |  | |  | | |
| 1 | 1(0.9) | | | 1(0.9) | | | |  | 2(4.3) | | | | |  | | | |  |  | | |  |  |  | |  |  |  |
| 2 |  | | |  | | | |  | 1(2.2) | | | | |  | | | |  |  | | |  |  |  | |  |  |  |
| 3 | 33(30.8) | | | 3(2.8) | | | |  | 12(26.1) | | | | | 2(4.3) | | | |  | 18(39.1) | | | 1(2.4) |  | 5(10.9) | |  |  |  |
| 4 | 72(67.3) | | | 80(74.8) | | | |  | 31(67.4) | | | | | 30(63.8) | | | |  | 28(60.9) | | | 34(81.0) |  | 40(87.0) | | 42(70.0) |  |  |
| 4a | 52(48.6) | | | 12(11.2) | | | |  | 26(56.5) | | | | | 7(14.9) | | | |  | 21(45.7) | | | 5(11.9) |  | 36(78.3) | | 7(11.7) |  |  |
| 4b | 17(15.9) | | | 24(22.4) | | | |  | 4(8.7) | | | | | 8(17.0) | | | |  | 6(13.0) | | | 13(31.0) |  | 2(4.3) | | 9(15.0) |  |  |
| 4c | 3(2.8) | | | 44(41.1) | | | |  | 1(2.2) | | | | | 15(31.9) | | | |  | 1(2.2) | | | 16(38.1) |  | 2(4.3) | | 26(43.3) |  |  |
| 5 | 1(0.9) | | | 22(20.6) | | | |  |  | | | | | 14(29.8) | | | |  |  | | | 7(16.7) |  |  | | 16(26.7) |  |  |
| 6 |  | | | 1(0.9) | | | |  |  | | | | | 1(2.1) | | | |  |  | | |  |  |  | | 2(3.3) |  |  |
| Stage |  | |  | |  | | | | |  | |  | | | |  | | | |  | | |  |  |  | | |  |
| 0 |  | | | 9(8.4) | | | |  |  | | | | | 3(6.4) | | | |  |  | | | 2(4.8) |  |  | | 5(8.3) |  |  |
| I |  | | | 35(32.7) | | | |  |  | | | | | 15(31.9) | | | |  |  | | | 14(33.3) |  |  | | 14(23.3) |  |  |
| II |  | | | 43(40.2) | | | |  |  | | | | | 22(46.8) | | | |  |  | | | 21(50.0) |  |  | | 37(61.7) |  |  |
| III |  | | | 18(16.8) | | | |  |  | | | | | 7(14.9) | | | |  |  | | | 5(11.9) |  |  | | 4(6.7) |  |  |
| IV |  | | | 2(1.9) | | | |  |  | | | | |  | | | |  |  | | |  |  |  | |  |  |  |
| Molecular Subtypes |  | |  | |  | | | | |  | |  | | | |  | | | |  | | |  |  |  | | |  |
| HER2+ |  | | | 26(24.3) | | | |  |  | | | | | 8(17.0) | | | |  |  | | | 15(35.7) |  |  | | 11(18.3) |  |  |
| HR+/HER2- |  | | | 63(58.9) | | | |  |  | | | | | 29(61.7) | | | |  |  | | | 20(47.6) |  |  | | 37(61.7) |  |  |
| TNBC |  | | | 13(12.1) | | | |  |  | | | | | 5(10.6) | | | |  |  | | | 3(7.1) |  |  | | 11(18.3) |  |  |
| Unknown |  | | | 5(4.7) | | | |  |  | | | | | 5(10.6) | | | |  |  | | | 4(9.5) |  |  | | 1(1.7) |  |  |
|  |  | | |  | | | |  |  | | | | |  | | | |  |  | | |  |  |  | |  |  |  |

**Table S11. Characteristics of the breast tissue samples in verifying the methylation model.**

|  | Training Set | | |  | Validation Set | | |
| --- | --- | --- | --- | --- | --- | --- | --- |
|  | Benign (n=28) | Malignant (n=105) | Total  (n=133) |  | Benign (n=28) | Malignant (n=104) | Total  (n=132) |
| Age | | | | | | | |
| 40-49 | 24(85.7%) | 45(42.9%) | 69(51.9%) |  | 20(71.4%) | 49(47.1%) | 69(52.3%) |
| 50-59 | 4(14.3%) | 50(47.6%) | 54(40.6%) |  | 8(28.6%) | 49(47.1%) | 57(43.2%) |
| ≥60 |  | 10(9.5%) | 10(7.5%) |  |  | 6(5.8%) | 6(4.5%) |
| ultrasonography BI-RADS | | | | | | | |
| 1 |  |  |  |  | 1(3.6%) |  | 1(0.8%) |
| 3 | 16(57.1%) | 2(1.9%) | 18(13.5%) |  | 8(28.6%) |  | 8(6.1%) |
| 4 | 12(42.9%) | 45(42.9%) | 57(42.9%) |  | 19(67.9%) | 49(47.1%) | 68(51.5%) |
| 4a | 11(39.3%) | 4(3.8%) | 15(11.3%) |  | 10(35.7%) | 4(3.8%) | 14(10.6%) |
| 4b | 1(3.6%) | 10(9.5%) | 11(8.3%) |  | 7(25.0%) | 3(2.9%) | 10(7.6%) |
| 4c |  | 26(24.8%) | 26(19.5%) |  | 2(7.1%) | 36(34.6%) | 38(28.8%) |
| 4 without ubcategory |  | 5(4.8%) | 5(3.8%) |  |  | 6(5.8%) | 6(4.5%) |
| 5 |  | 57(54.3%) | 57(42.9%) |  |  | 55(52.9%) | 59(44.7%) |
| 6 |  | 1(1.0%) | 1(0.8%) |  |  |  |  |
| Stage |  |  |  |  |  |  |  |
| 0 |  |  |  |  |  | 2(1.9%) | 2(1.5%) |
| I |  | 28(26.7%) | 28(21.1%) |  |  | 24(23.1%) | 24(18.2%) |
| II |  | 47(44.8%) | 47(35.3%) |  |  | 56(53.8%) | 56(42.4%) |
| III |  | 30(28.6%) | 30(22.6%) |  |  | 22(21.2%) | 22(16.7%) |
| Molecular Subtypes |  |  |  |  |  |  |  |
| HER2+ |  | 43(41.0%) | 43(32.3%) |  |  | 46(44.2%) | 46(34.8%) |
| HR+/HER2- |  | 45(42.9%) | 45(33.8%) |  |  | 43(41.3%) | 43(32.6%) |
| TNBC |  | 17(16.2%) | 17(12.8%) |  |  | 15(14.4%) | 15(11.4%) |
